# Taeniasis and cysticercosis due to *Taenia solium *in Japan

**DOI:** 10.1186/1756-3305-5-18

**Published:** 2012-01-17

**Authors:** Tetsuya Yanagida, Yasuhito Sako, Minoru Nakao, Kazuhiro Nakaya, Akira Ito

**Affiliations:** 1 Asahikawa Medical University, Asahikawa, Hokkaido, 078-8510, Japan

**Keywords:** *Taenia solium*, Cysticercosis, Taeniasis, Japan

## Abstract

*Taenia solium *is a zoonotic cestode that causes taeniasis and cysticercosis in humans. The parasite is traditionally found in developing countries where undercooked pork is consumed under poor sanitary conditions and/or as part of traditional food cultures. However, the recent increase in international tourism and immigration is spreading the disease into non-endemic developed countries such as the United States. Although there has been concern that the number of cysticercosis cases is increasing in Japan, the current situation is not clear. This is largely because taeniasis and cysticercosis are not notifiable conditions in Japan and because there have been no comprehensive reviews of *T. solium *infections in Japan conducted in the last 15 years. Herein, we provide an overview of the status of *T. solium *infection in Japan over the past 35 years and point out the potential risks to Japanese society.

## Review

*Taenia solium *is a cestode species that causes taeniasis and cysticercosis in humans. Taeniasis refers to the intestinal infection with the adult stage of the tapeworm, which is acquired by eating undercooked pork contaminated with cysticerci (larval stage). On the other hand, cysticercosis refers to infection of various tissues with the larval stage of the tapeworm (cysticercus/cysticerci), which is acquired by ingestion of parasite eggs released from taeniasis carriers. Whilst taeniasis is asymptomatic in many carriers, neurocysticercosis (NCC), cysticercosis of the central nervous system, is still often lethal especially in remote areas in developing countries [1-3]. Although cysticercosis is one of the most important cestode zoonoses worldwide [4,5], it is endemic mainly in remote or rural areas of developing countries where local people consume pork without the benefit of meat inspection. However, recent trends in international tourism into remote or rural areas, expansion of global business and an increase in immigrants and refugees have increased the number of taeniasis and cysticercosis cases in the developed country such as the United States [6,7]. Globalization has even resulted in taeniasis and cysticercosis cases being identified in communities where eating pork is prohibited by religious law [6,8]. Some of these cases are believed to have occurred due to fecal-oral transmission of parasite eggs shed in the feces of housekeepers or food handlers who emigrated from endemic countries and harbored adult worms. Also, transmission is facilitated by poor or no knowledge of the risk of infection through consumption of local foods while visiting endemic countries. To prevent further outbreaks of cysticercosis in developed countries, the establishment of central or local governmental surveillance systems is an urgent task. In these systems, it is essential to introduce highly reliable detection tools and sustainable education programs. Molecular detection of taeniasis is highly reliable for differentiation of *T. solium *from other *Taenia *species in human stool specimens [1-4]. It is also available for histopathological specimens from cysticercosis patients [9-12]. Genotyping of mitochondrial DNA (mtDNA) enable us to assess where the infection was acquired from [9,10]. In contrast, serology is important for confirmation of cysticercosis in patients with suspected NCC based on neuroimaging [2,11].

At present, *T. solium *infection is not endemic in Japan and cysticercosis cases have been reported only sporadically. However, in the last 15 years, the number of foreign residents in Japan has risen from 1.4 to 2.2 million, and Japanese nationals staying abroad for a prolonged period of time (> 3 months) has also increased from 430,000 to 760,000 (Ministry of Foreign Affairs of Japan, 2010). Although greater movement of people has possibly increased the number of *T. solium *infections in Japan, the actual number of the cases is largely unknown as taeniasis and cysticercosis are not notifiable in Japan. In addition, a recent outbreak of human taeniasis due to *Taenia asiatica*, which also requires pig as the intermediate host, in Tokyo, Japan [13] prompts us to reevaluate the risk of *T. solium *infection in Japan. The aim of this study was, therefore, to grasp and summarize the current status of *T. solium *infection in Japan. In the present study, we review case reports published in Japan during the past 17 years, and report briefly on 3 previously unpublished cysticercosis cases, which can indicate the present situation and level or reliability in diagnosis of cysticercosis in Japan. The three new cysticercosis case reports were diagnosed using neuroimaging and confirmed with immunoblot and enzyme-linked immunosorbent assay (ELISA) using recombinant chimeric antigens [14]. These individuals were also tested for taeniasis using multiplex polymerase chain reaction (PCR) [15] and loop-mediated isothermal amplification (LAMP) [16].

## Literature review

The PubMed [17] and Ichushi-Web [18] databases were searched for papers and reports on cysticercosis in Japan from 1994 through 2010. In both databases, the words "*Taenia solium*", "cysticercosis" and "Japan" were used as the search terms. For comparison, summarized data on cysticercosis cases in Japan from 1976 to 1993 were evaluated from a previous review [19]. Table 1 summarizes the differences in the frequency of cysticercosis cases in the investigated periods. In 1994-2010, 39 cases (27 papers and 12 abstracts of conference presentations) were found (2.3 cases/year) and the incidence of reported cases was similar in 1976-1993 (2.4 cases/year). The male-to-female ratio in 1994-2010 was significantly lower than in 1976-1993 (P < 0.05, Fisher's exact test). There was a recent tendency showing a wider generation including children and a larger number of women suffering from cysticercosis. In 1994-2010, 22 patients were Japanese (56.4%), 11 patients were from Asian countries including China (n = 6), Korea, Cambodia, Nepal, India and the Philippines, and 2 others were from Latin America (Brazil and Peru). The nationality was not provided for the remaining 4 cases. Among the Japanese patients whose travel histories could be ascertained, 13 patients visited Asian countries while only one visited Latin America. We have to stress that 7 patients had no history of travel abroad. In 1994-2010, there were 35 NCC, 7 subcutaneous (or muscular) (SCC), and 2 ocular cysticercosis (OCC) cases. Some patients had NCC plus SCC (n = 4) and NCC plus OCC (n = 1). Only one taeniasis case was reported as a concomitant infection with NCC. There was a crucial difference in the treatment of cysticercosis cases. When cysticercosis cases were diagnosed by serologic tests in advance, 78.6% (11/14) were treated only with chemotherapy, and 21.4% (3/14) were treated surgically. By contrast, only 12% of cysticercosis cases (3/25) either sero-negative and/or no serologic test applied for were treated with chemotherapy, but 88% (22/25) of them were treated surgically, often being misdiagnosed as having a brain tumor [20-22].

**Table 1:** Summarized data on cysticercosis cases reported in Japan between 1976-1993 and 1994-2010.

	1976-1993	1994-2010
No. cases*	43 (2.4)	39 (2.3)
Sex (% Male)	71.8	43.6
Age**	ND (20-62)	39.9 (9-70)
Japanese (%)	60.5	56.4
E Asia (%)***	25.6	17.9

*The total number and incidence (number of cases/year) of cases. **The mean and range (in parentheses) of patient ages. ***Percentage of East Asian patients. ND: no data.

## Case reports

### Case 1

In 2005, a 42-year-old Brazilian woman presented to an eye doctor with a visual impairment, and pappiledema. Upon neurological examination, she was diagnosed with communicating hydrocephalus. A ventriculo-peritoneal shunt was implanted and the hydrocephalus improved. Neuroendoscopic surgery showed multiple cysts in the subarachnoidal space, and histopathological examination indicated cestode-derived tissue. Serology for cysticercosis was strongly positive, but copro-multiplex PCR was negative for taeniasis. The patient was treated with praziquantel at first and then with albendazole. Post-treatment serology remained positive.

### Case 2

A 38-year-old Japanese man expelled a single tapeworm in 2000. He was treated with pyrantel pamoate, a drug used primarily for nematode parasites. In 2004, he complained of headaches and multiple cystic lesions were found on brain computed tomography (CT). However, at this time, no medical or surgical treatment was initiated because all of the cysts were deemed dead based on the CT image and the patient's normal eosinophil value. In 2009, he complained of a narrowing field of vision. Serology showed strong positive responses, but copro-LAMP was negative. The patient was treated with albendazole in 2010, and follow-up is ongoing.

### Case 3

A 31-year-old Japanese man noticed a subcutaneous nodule on his neck in December 2009. When the number of nodules increased by May 2010, brain CT and magnetic resonance imaging (MRI) showed multiple cystic lesions in the brain and serology showed strong positive responses. Tapeworm proglottids were expelled in his stools at the beginning of July 2010 and were identified as *T. solium *by multiplex PCR. He remembered he had found a similar white noodle-like substance in 2009, but he did not care about it. Taeniasis was treated using Gastrografin (diatrizoate meglumine and diatrizoate sodium) at Mie University hospital in Mie, Japan on 14 July, but no worms were expelled. However, *T. solium *infection was confirmed by copro-LAMP and *Taenia *eggs were identified microscopically. On July 28th 2010, the patient expelled additional worms. Then he was treated with Gastrografin and albendazole in August 2010, but tapeworm(s) were not expelled. However, it was considered that the patient became free from *T. solium *tapeworm after he found the worm by himself on 28th July, as copro-LAMP was negative until December 2010. In contrast, the subcutaneous and intracranial nodules disappeared within 4 weeks after the treatment.

The patient's travel history indicated that he had worked for one month in a rural area 150 km from Delhi, India on 3 separate occasions. The first, second and third visits were March-April 2008, October-November 2008 and March-April 2010, respectively. Based on this information, it was suspected that he acquired the parasite during his first or second stay in India. A mtDNA gene (chromosome *c *oxidase subunit 1) of the obtained proglottids was sequenced according to previous research [23], and was determined to be identical to the Indian haplotype of *T. solium *(GenBank Accession Number: AB066489). Due to the risk of the patient contaminating his living environment with parasite eggs while infected with the intestinal form of *T. solium*, his wife and 4 year old son were tested serologically for cysticercosis. In addition, 41 colleagues who had also travelled to India since 2007 were tested serologically for cysticercosis, with 39 of these individuals also tested for taeniasis using copro-LAMP. To date, none of these individuals have tested positive for either cysticercosis or taeniasis. This case is notable as a rare imported case of *T. solium *taeniasis/cysticercosis in Japan.

## Discussion

*T. solium *infection tends to be endemic in developing countries, because the main risk factors for infection are poor sanitation and free-roaming pig farming [24]. In Japan, the number of cysticercosis cases was highest during World War II (225 cases during 1936-1945) and then drastically decreased by 1994 (15 cases during 1986-1993) [19]. However, cysticercosis is now considered as an emerging public health problem in some developed countries mainly USA [6,7,25] and also in Canada [2,26] and Europe where pig farming style has changed from indoor to outdoor with immigrants from Latin America etc. [27-29]. Although it has become apparent that an increase in international travel raises the risk of importation of *T. solium *into Japan, the incidence of reported cases was almost the same in the current review (2.4 per year; 1994-2010) as in the previous review (2.3 per year; 1976-1993) [19]. However, the actual number of cases is most likely underestimated, as taeniasis and cysticercosis are not notifiable in Japan. To clarify the present situation of *T. solium *infection in Japan, advanced diagnostic tools should be effectively utilized under governmental recommendations. Also, *T. solium *infection should be included on Japan's list of notifiable infectious diseases.

Our laboratory in Asahikawa Medical University has been an exclusive reference laboratory for serodiagnosis in Japan from 1998 when we reported serologic data [14,30-34]. We have been involved in many bi- and multi-lateral projects on taeniasis and cysticercosis mainly in Asia (China, Vietnam, Philippines, Thailand, Lao PD, Nepal, Indonesia, Papua New Guinea, India) and some in Africa (South Africa, Mozambique, Tanzania, Cameroon, Ethiopia) [35-43]. Based on our own experience and from many other studies by other groups on the transmission studies of cysticercosis, it is possible that a single taeniasis patient or carrier can contaminate the living environment with parasite eggs and infect numerous others with cysticercosis. Thus, follow-up studies using immunological tests are necessary not only for the taeniasis patients but also for their family members, neighbours, and close relatives. Despite the fact that autochthonous cysticercosis has not been confirmed recently in Japan, 7 cysticercosis cases were identified without a history of travel abroad. Therefore, it is hypothesized that these cases acquired the infection from unknown carriers in Japan. MtDNA analysis using stool samples, eggs, proglottids or metacestodes is highly useful to assess where the infection was acquired [9-11,15,42].

In the United States, 3,937 NCC hospitalizations were identified in Los Angeles County from 1991 through 2008, and more than 90% of the patients were Latinos [44]. Other reports have also demonstrated that most of the NCC patients in the US were Hispanics that emigrated from endemic countries such as Mexico [6,45,46]. Although it has been considered that most of the NCC patients had been infected in their homelands, a recent study conducted along the US-Mexico border revealed that the prevalence of taeniasis was 3% [47], indicating a certain number of *T. solium *taeniasis carriers are also crossing the border. A similar situation between Myanmar and Thailand has recently been reported [35]. In this study, approximately 3.3% (5/150) of refugees from Myanmar in a refugee village, Tak Province, Thailand, were confirmed to have *T. solium *taeniasis in February 2011. However, when the Thai group members re-visited the village in May 2011 to provide treatment to the taeniasis cases, 3 of the patients had gone to Bangkok before they could be treated (Kusolsuk et al. in prep.).

In contrast to the US, the majority of foreign residents in Japan are not Hispanic. There were approximately 2.2 million foreign residents in Japan in 2009, and 16% of them were Latin Americans, while 77% were Asians (The Ministry of Justice, Japan, 2010). From 1976 to 1993, cysticercosis was reported in 12 foreign residents in Japan, with 11 of the cases from East Asian countries including China and North and South Korea. However, the current review of the literature indicates an increasing risk of importing the disease from South and Southeast Asian countries. From 1994 to 2010, there were 10 Japanese female patients whose travel histories could be ascertained, with 7 of these patients having traveled to endemic Asian countries such as Thailand, India, Nepal and Vietnam. Also, 4 NCC have been reported in foreign residents from India, Nepal, Cambodia and the Philippines. Based on mtDNA analysis of proglottids and on travel histories, it was concluded that infections in 2 of the Japanese cases were acquired in Nepal [9] and India (Case 3) during business trips. The former case was suspected to be NCC by neurologists but serology carried out in the hospital was negative. However, serology using specific diagnostic antigens carried out in Asahikawa was positive before surgery but negative one year after surgery [21].

In 2010, more than 20 taeniasis cases caused by *Taenia asiatica*, another human-infecting *Taenia *species which requires pigs as the intermediate host, were reported from Tokyo and surrounding areas [13]. *T. asiatica *is endemic in several Asian countries, such as China, Taiwan, Korea, Indonesia, Vietnam, the Philippines and Thailand where local people eat uncooked pig liver [1,33,39-41,43]. In Japan, 2 taeniasis cases in 1968 and 1992 were retrospectively analyzed and the expelled worms were confirmed as *T. asiatica*, [48,49] but there was no crucial evidence of autochthonous infection. However, the taeniasis patients in the 2010 report [13] had no travel history to known endemic countries during the last decade, but instead ate raw indigenous pork or beef livers (sliced liver "liver sashimi") in Tokyo and surrounding areas. Therefore, these are considered to be cases of autochthonous taeniasis caused by contaminated domestic animals. A possible source of contamination is tapeworm carriers working on Japanese pig or cattle farms. Recently, foreign agricultural workers from Asian developing countries are common not only in Japan but also in Europe [50,51] and New Zealand [52]. It is probable that at least some of these individuals are infected with tapeworms and are responsible for contaminating farming areas. This is especially likely since there are no regulations in place to check visitors to Japan for anything other than diarrhea or fever. Under the current conditions, it is likely that an outbreak of *T. solium *infection will also occur in the near future.

In our literature review for the years 1994-2010, there was a significant correlation between the types of treatment received and immunological diagnosis. In most of the patients who received no immunological testing, mainly serology or showed negative responses on immunodiagnosis, cysts were misdiagnosed as brain tumors and surgically resected, whereas the majority of patients who were serologically confirmed to have cysticercosis received only chemotherapy. To avoid unnecessary operations, reliable serologic examinations should be applied to patients with suspicious neuroimaging results. However, NCC may not be suspected by clinicians working in non-endemic areas due to the lack of knowledge or experience about this disease. Another example of inadequate treatment was found in Case 2. This patient was treated with pyrantel pamoate, a drug used for nematodiasis, despite a diagnosis of cestodiasis. Therefore, education on parasitic diseases should be strengthened for clinicians and for the general public.

## Conclusions

Although the incidence of cysticercosis in Japan has seemingly not increased over the past 35 years, diversification of the profile of patients was observed, such as an increase in the percentage of female patients, wider age distributions and the risk of importing the disease from South and Southeast Asia. This change is likely the result of the globalization of business and increased tourism to remote areas in developing countries. Another important risk is the employment of foreign workers in agriculture. The agricultural population in Japan has been decreasing and therefore, immigrant labour from developing countries is becoming essential. To control this neglected parasitic disease in Japan, recognition of the possible risks is necessary. In addition, it would be desirable to include *T. solium *infection on Japan's list of notifiable diseases. Cooperation among clinicians, researchers and governmental officials is needed to address the prevention of the introduction of *T. solium *into Japan.

## Competing interests

The authors declare that they have no competing interests.

## Authors' contributions

TY carried out the molecular diagnosis and analysis of the taeniasis/cysticercosis cases and drafted the manuscript and revised it. YS carried out immunological examination on the cases. MN carried out some of the molecular analysis. KN carried out histopathology of the cases. AI participated in designing the study and helped to draft the manuscript and revised it. All authors read and approved the final manuscript. Core MS was prepared based on TY's talk entitled "Taeniasis/cysticercosis of *Taenia solium *in developed countries introduced by people from developing countries: some Japanese cases" at the JSPS-AASP Symposium "Toward the prevention of taeniasis and cysticercosis in Asia: emergent problems from developing to developed countries" on 3 Dec 2010 at the Joint International Tropical Medicine Meeting 2010 (JITMM2010), Bangkok, Thailand. All authors read and approved the final manuscript.

## References

[B1] Ito A Nakao M Wandra T Human taeniasis and cysticercosis in Asia Lancet 2003 362 1918 1920 10.1016/S0140-6736(03)14965-3 14667751

[B2] Schantz PM Wilkins PP Tsang VCW Scheld WW, Craig WA, Hughes JM Immigrants, imaging and immunoblots: the emergence of neurocysticercosis as a significant public health problem Emerging Infections 2 1998 Washington DC: ASM Press 213 242

[B3] Flisser A Craig PS Ito A Palmer SR, Lord Soulsby, Torgerson PR, Brown DWG Cysticercosis and taeniosis: *Taenia solium, Taenia saginata *and *Taenia asiatica* Oxford Textbook of Zoonoses 2011 Oxford: Oxford University Press 625 642

[B4] Dorny P Praet N Deckers N Gabriel S Emerging food-borne parasites Vet Parasitol 2009 163 196 206 10.1016/j.vetpar.2009.05.026 19559535

[B5] Molyneux D Hallaj Z Keusch GT McManus DP Ngowi H Cleaveland S Ramos-Jimenez P Gotuzzo E Kar K Sanchez A Garba A Carabin H Bassili A Chaignat CL Meslin FX Abushama HM Willingham AL Kioy D Zoonoses and marginalised infectious diseases of poverty: Where do we stand? Parasit Vectors 2011 4 106 10.1186/1756-3305-4-106 21672216 PMC3128850

[B6] Schantz PM Moore AC Muñoz JL Hartman BJ Schaefer JA Aron AM Perasaud D Sarti E Wilson M Flisser A Neurocysticercosis in an Orthodox Jewish community in New York City N Engl J Med 1992 327 692 695 10.1056/NEJM199209033271004 1495521

[B7] Sorvillo F Wilkins P Shafir S Eberhard M Public health implications of cysticercosis acquired in the United States Emerg Infect Dis 2011 17 1 6 21192847 10.3201/eid1701.101210 PMC3298370

[B8] Hira PR Francis I Abdella NA Gupta R Al-Ali FM Grover S Khalid N Abdeen S Iqbal J Wilson M Tsang VCW Cysticercosis: imported and autochthonous infections in Kuwait Trans Roy Soc Trop Med Hyg 2004 98 233 239 10.1016/S0035-9203(03)00061-0 15049462

[B9] Yanagida T Yuzawa I Joshi DD Sako Y Nakao M Nakaya K Kawano N Oka H Fujii K Ito A Neurocysticercosis: assessing where the infection was acquired from J Trav Med 2010 17 206 208 10.1111/j.1708-8305.2010.00409.x 20536894

[B10] Jongwutiwes U Yanagida T Ito A Kline SE Isolated intradural-extramedullary spinal cysticercosis: a case report J Trav Med 2011 18 284 287 10.1111/j.1708-8305.2011.00535.x 21722242

[B11] Ito A Takayanagui OM Sako Y Sato MO Odashima NS Yamasaki H Nakaya K Nakao M Neurocysticercosis: clinical manifestation, neuroimaging, serology and molecular confirmation of histopathologic specimens Southeast Asian J Trop Med Public Health 2006 27 Suppl 3 74 81 17547057

[B12] Yamasaki H Nakao M Sako Y Nakaya K Ito A Molecular identification of *Taenia solium *cysticercus genotype in the histopathological specimens Southeast Asian J Trop Med Public Health 2005 36 Suppl 131 134 16438197

[B13] Yamasaki H Muto M Morishima Y Sugiyama H Kawanaka M Nakamura-Uchiyama F Ohgame M Kobayashi K Ohnishi K Kawai S Okuyama T Saito K Miyahira Y Yanai H Matsuoka H Haruki K Miyoshi Y Akao N Akiyama J Araki J *Taenia asiatica *infection occurring in Kanto District, 2010 [in Japanese] Clin Parasitol 2011 22 75 78

[B14] Sako Y Nakao M Ikejima T Piao XZ Nakaya K Ito A Molecular characterization and diagnostic value of *Taenia solium *low-molecular-weight antigen genes J Clin Microbiol 2000 38 4439 4444 11101577 10.1128/jcm.38.12.4439-4444.2000 PMC87618

[B15] Yamasaki H Allan JC Sato MO Nakao M Sako Y Nakaya K Qiu DC Mamuti W Craig PS Ito A DNA differential diagnosis of taeniasis and cysticercosis by multiplex PCR J Clin Microbiol 2004 42 548 553 10.1128/JCM.42.2.548-553.2004 14766815 PMC344500

[B16] Nkouawa A Sako Y Nakao M Nakaya K Ito A Loop-mediated isothermal amplification method for differentiation and rapid detection of *Taenia *species J Clin Microbiol 2009 47 168 174 10.1128/JCM.01573-08 19005142 PMC2620829

[B17] PubMed http://www.ncbi.nlm.nih.gov/pubmed/

[B18] Ichushi-Web, Japan Medical Abstract Society http://search.jamas.or.jp/

[B19] Araki T President lecture: current trend of cerebral cysticercosis in Japan Clin Parasitol 1994 5 12 24 (in Japanese)

[B20] Ohsaki N Matsumoto A Miyamoto K Kondoh N Araki K Ito A Kikuchi K Neurocysticercosis without detectable specific antibody Internal Med 1999 38 67 70 10.2169/internalmedicine.38.67 10052747

[B21] Ito A Nakao M Ito Y Yuzawa I Morishima H Kawano N Fujii K Neurocysticercosis case with a single cyst in the brain showing dramatic drop in specific antibody titers within 1 year after curative surgical resection Parasitol Int 1999 48 95 99 10.1016/S1383-5769(99)00005-7 11269331

[B22] Ishikawa E Komatsu Y Kikuchi K Yamasaki H Kimura H Osuka S Tsurubuchi T Ito A Matsumura A Neurocysticercosis as solitary parenchymal lesion confirmed by mitochondrial deoxyribonucleic acid sequence analysis Neuro Med Chir (Tokyo) 2007 47 40 44 10.2176/nmc.47.40 17245015

[B23] Nakao M Okamoto M Sako Y Yamasaki H Nakaya K Ito A A phylogenetic hypothesis for the distribution of two genotypes of the pig tapeworm *Taenia solium *worldwide Parasitology 2002 124 657 662 12118722 10.1017/s0031182002001725

[B24] Enander RT Amaya AR Enander RA Gute DM Neurocysticercosis: risk and primary prevention strategies update Int J Environ Health Res 2010 20 329 365 10.1080/09603123.2010.482152 20853197

[B25] del La Garza Y Graviss EA Daver NG Gambarin KJ Shandera WX Schantz PM White AC Jr Epidemiology of neurocysticercosis in Houston, Texas Am J Trop Med Hyg 2005 73 766 770 16222023

[B26] Burneo JG Plener I Garcia HH Neurocysticercosis in a patient in Canada Canadian Med Assoc J 2009 180 639 642 10.1503/cmaj.081452 PMC2653585 19289809

[B27] Esquivel A Diaz-Otero F Gimenez-Roldan S Growing frequency of neurocysticercosis in Madrid (Spain) Neurologia 2005 20 116 120 15815946

[B28] Waloch M Cestode infections in Poland 2007 Przegl Epidemiology 2009 63 267 9 (in Polish) 19799259

[B29] Croft AM Flores AA López HZ Cysticercosis in a female Nicaraguan traveler J Travel Med 2007 14 349 351 10.1111/j.1708-8305.2007.00149.x 17883469

[B30] Ito A Plancarte A Ma L Kong Y Flisser A Cho SY Liu YH Kamhawi S Lightowlers MW Schantz PM Novel antigens for neurocysticercosis: simple method for preparation and evaluation for serodiagnosis Am J Trop Med Hyg 1998 59 291 294 9715949 10.4269/ajtmh.1998.59.291

[B31] Ito A Serologic and molecular diagnosis of zoonotic larval cestode infections Parasitol Int 2002 51 221 235 10.1016/S1383-5769(02)00036-3 12243777

[B32] Ito A Sako Y Yamasaki H Mamuti W Nakaya K Nakao M Ishikawa Y Development of Em18-immunoblot and Em-18-ELISA for specific diagnosis of alveolar echinococcosis Acta Trop 2003 85 173 182 10.1016/S0001-706X(02)00221-8 12606094

[B33] Ito A Craig pS Immunodiagnostic and molecular approaches for the detection of taeniid cestode infections Trends Parasitol 2003 19 377 381 10.1016/S1471-4922(03)00200-9 12957509

[B34] Deckers N Dorny P Immunodiagnosis of *Taenia solium *taeniasis/cysticercosis Trends Parasitol 2010 26 137 144 10.1016/j.pt.2009.12.008 20083438

[B35] Ito A Okamoto M Li T Wandra T Dharmawan NS Swastika KI Dekumyoy P Kusolsuk T Davvajav A Davvasuren A Dorjsuren T Mekonnen SM Hegasi ZH Yanagida T Sako Y Nakao M Nakaya K Lavikainen AJ Nkouawa A Mohammadzadeh T The first workshop towards the control of cestode zoonoses in Asia and Africa Parasit Vectors 2011 4 114 10.1186/1756-3305-4-114 21693001 PMC3141742

[B36] Ito A Okamoto M Wandra T Wibisono H Anantaphruti MT Waikagul J Li T Qiu D The present situation of taeniasis and cysticercosis in Asia and the Pacific Southeast Asian J Trop Med Public Health 2007 38 Suppl 1 119 124

[B37] Ikejima T Piao ZX Sako Y Sato MO Bao S Si R Yu F Zhang CL Nakao M Yamasaki H Nakaya K Ito A Evaluation of clinical and serological data from *Taenia solium *cysticercosis patients in eastern Inner Mongolia Autonomous region, China Trans R Soc Trop Med Hyg 2005 99 625 630 10.1016/j.trstmh.2005.04.008 15927218

[B38] Tran DS Odermatt P Le Oanh T Huc P Phoumindr N Ito A Druet-Cabanac M Perux PM Strobel M Risk factors for epilepsy in rural Lao PDR: a case-control study Southeast Asian J Trop Med Public Health 2007 38 537 42 17877231

[B39] Ito A Craig PS Schantz PM Taeniasis/cysticercosis and echinococcosis with focus on Asia and the Pacific Parasitol Int 2006 55 S1 S308 16343981 10.1016/j.parint.2005.11.001

[B40] Li T Craig PS Ito A Chen X Qiu D Qiu J Sato MO Wandra T Bradshaw H Li L Yang Y Wang Q Taeniasis/cysticercosis in a Tibetan population in Sichuan province, China Acta Trop 2006 100 223 231 10.1016/j.actatropica.2006.11.003 17166477

[B41] Anantaphruti MT Yamasaki H Nakao N Waikagul J Wattanakulanich D Nuamtanong S Maipanich W Pubampen S Sanguankiat S Muennoo C Nakaya K Sato MO Sako Y Okamoto M Ito A Sympatric occurrence of *Taenia solium, T. saginata*, and *T. asiatica*, Thailand Emerg Infect Dis 2007 13 1413 1416 18252126 10.3201/eid1309.061148 PMC2857269

[B42] Swastika K Dewiyani CI Yanagida T Sako Y Sudamaja M Sutisna P Wandra T Dharmawan NS Nakaya K Okamoto K Ito A An ocular cysticercosis in Bali, Indonesia caused by *Taenia solium *Asian genotype Parasitol Int 2011 10.1016/j.parint.2011.11.004 22146156

[B43] Somers R Dorny P Geysen D Nguyen LA Thach DC Vercruysse J Ngouyen VK Human tapeworms in north Vietnam Trans R Soc Trop Med Hyg 2007 101 275 277 10.1016/j.trstmh.2006.04.007 16806333

[B44] Croker CC Reporter R Mascola L Use of statewide hospital discharge data to evaluate the economic burden of neurocysticercosis in Los Angeles County Am J Trop Med Hyg 2010 83 106 110 10.4269/ajtmh.2010.09-0494 20595487 PMC2912585

[B45] Townes JM Hoffman CJ Kohn MA Neurocysticercosis in Oregon, 1995-2000 Emerg Infect Dis 2004 10 508 510 15109424 10.3201/eid1003.030542 PMC3322801

[B46] Wallin MT Kurtzke JF Neurocysticercosis in the United States. Review of an important emerging infection Neurology 2004 63 1559 1564 15534236 10.1212/01.wnl.0000142979.98182.ff

[B47] Barton Behravesh C Mayberry LF Bristol JR Cardenas VM Mena KD Martínez-Ocaña J Flisser A Snowden KF Population-based survey of taeniasis along the United States-Mexico border Ann Trop Med Parasitol 2008 102 325 333 10.1179/136485908X300788 18510813

[B48] Eom KS Jeon HK Rim HJ Geographic distribution of *Taenia asiatica *and related species Korean J Parasitol 2009 47 S115 S124 10.3347/kjp.2009.47.S.S115 19885327 PMC2769216

[B49] Jeon HK Kim KH Eom KS Molecular identification of *Taenia *specimens after long-term preservation in formalin Parasitol Int 2011 60 203 205 10.1016/j.parint.2010.12.001 21163367

[B50] Dorny P Praet N *Taenia saginata *in Europe Vet Parasitol 2007 149 22 24 10.1016/j.vetpar.2007.07.004 17706360

[B51] Flutsch F Heinzmann D Mathis A Herzberg H Stephan R Deplazes P Case-control study to identify risk factors for bovine cysicercosis on farms in Switzerland Parasitology 2008 135 641 646 18371237 10.1017/S0031182008004228

[B52] McFadden AM Heath DD Morley CM Dorny P Investigation of an outbreak of *Taenia saginata *cysts (*Cysticercus bovis*) in dairy cattle from two farms Vet Parasitol 2011 176 177 184 10.1016/j.vetpar.2010.10.058 21130574

